# Acetonic Fraction of *Bidens pilosa* Enriched for Maturase K Is Able to Control Cerebral Parasite Burden in Mice Experimentally Infected With *Toxoplasma gondii*

**DOI:** 10.3389/fvets.2019.00055

**Published:** 2019-03-06

**Authors:** Caroline Martins Mota, Fernanda Maria Santiago, Mariana de Resende Damas Cardoso, Cristina Rostkowska, Taísa Carrijo de Oliveira, Deise Aparecida de Oliveira Silva, Carlos Priminho Pirovani, Tiago Wilson Patriarca Mineo, José Roberto Mineo

**Affiliations:** ^1^Laboratory of Immunoparasitology “Dr. Mário Endsfeldz Camargo”, Institute for Biomedical Sciences, Federal University of Uberlândia, Uberlândia, Brazil; ^2^Department of Biological Sciences, Biotechnology and Genetic Center, Santa Cruz State University, Ilhéus, Brazil

**Keywords:** *Bidens pilosa*, total and acetonic extracts, *Toxoplasma gondii*, maturase k, infection control

## Abstract

*Toxoplasma gondii* infection can cause abortions or congenital infection for a vast number of domestic animals and humans, leading to economic loss in veterinary sciences, as well as severe consequences for immunocompromised patients. *Bidens pilosa* Linné has been used in ethnopharmacology for treatment of diseases, as malaria, diabetes and hepatitis, in addition to its use as antioxidant, antiallergic, anti-inflammatory, and antiviral. The components of this plant have never been studied before for treatment of toxoplasmosis, and the conventional drugs currently used to treat this disease have high degree of toxicity. Thus, the aim of this study was to evaluate the effect of *B. pilosa* against *T. gondii*, by analyzing a total extract of this plant in parallel with a fraction obtained by precipitation in acetone. Also, it was assessed if the acetonic fraction could present lectinic activity, followed by its identification by mass spectrometry. It was observed with the experimental models designed that both total extract and acetonic fraction of *B. pilosa* were able to control *T. gondii* infection by *in vitro* and *in vivo* experiments, in addition to their low toxicity to host cells. Both total extract and acetonic fraction of this plant display capacity to impair replication of *T. gondii* tachyzoites. Interesting, the *B. pilosa* acetonic fraction treatment for 10 days after infection decreases significantly the number of *T. gondii* brain cyst in comparison with controls. The protein isolated from *B. pilosa* acetonic fraction was characterized as a novel lectin identified as maturase K. Taken together, these findings open new perspectives to treat patients infected by *T. gondii*. Future studies will be necessary to investigate the precise mechanism underlying the control of *T. gondii* infection to impair the replication of this parasite in the host cells after treatment with *B. pilosa* maturase K.

## Introduction

*Toxoplasma gondii* is an obligate intracellular protozoan member of the Apicomplexa phylum displaying zoonotic characteristics and a heteroxenic life cycle accomplishing a sexual and asexual mode of replication. The sexual replication occurs in the intestine of felids, its definitive hosts, and produces oocysts, which are excreted and undergo meiosis and produce sporozoites. Once ingested by intermediate hosts, a vast number of domestic animals and humans, these parasites convert to the tachyzoite stage, which is responsible to induce toxoplasmosis (1). The infection occurs mainly via oral by ingestion of oocysts from the environment, but consumption of raw or undercooked meat containing tissue cysts may also lead to infection. In addition, the parasite can reach the fetal tissues by crossing placental barrier, particularly when the maternal organism has no protective immune response (2, 3). It has been described in the literature an increasing rates of *T. gondii* tissue cysts in meat-producing animals, as well as the prevalence of oocysts released by cats on the environment, increasing the infection-risk for domestic animals and human population (2, 3).

*T. gondii* is a protozoan parasite that infects about 2–3 billion people worldwide (4). Infection by *T. gondii* is usually asymptomatic in immunocompetent individuals, but can cause abortions or congenital infections in immunocompetent individuals and severe consequences in immunocompromised patients (5–7). Currently, there is no available drug able to eliminate the parasite, even though there are drugs that can impair the multiplication of the parasite during its active stage of replication. However, once the parasite encysts in the tissues, these drugs lose their effectiveness (8–10). Thus, the challenge is the characterization of new drugs to treat *T. gondii* infection, considering that the drugs currently used are not totally effective, as well as their degree of toxicity or hypersensitivity for many patients are undesirable and require prolonged courses (8, 11, 12). The novel drugs against *T. gondii* infection, including those having the cyst stage as target have been already described in the literature, but all of them need additional evidences to be used in patients, as they are still in preclinical phase. In fact, there are over 20 preclinical drug development projects that have been described in publications over the past two decades. In this context, the basic research in *T. gondii* biology will make possible to identify a diverse array of drug targets, as the current investigations of drug targets in *T. gondii* that has recently been advanced by using CRISPR/Cas9 genome-wide screen to discover additional essential genes (8).

*Bidens pilosa* Linné, which belongs to the Asteraceae family, has been used in ethnopharmacology for many years and nowadays is widely studied to treat certain diseases, such as malaria, diabetes, and hepatitis, in addition to its use as antioxidant, antiallergic, anti-inflammatory, and antiviral effects (12–18). Also, no further characterization has been published concerning the molecular features from *B. pilosa*, i.e., the presence of enzymes displaying critical functions or additional actions, as affinity by carbohydrates. In this context, lectins from plants has been described in the literature as potentials immunological tools to control parasite infections caused by *Leishmania* or *Neospora caninnun*, as well as adjuvants in vaccination protocols (15, 16, 19). Lectins are proteins with capacity to bind specifically to carbohydrates and can be isolated from many different sources, including plant and animal tissues (19). These proteins are essential to diverse intracellular processes, such as interactions among different cells and extracellular matrix, cell adhesion and migration, embryogenesis, and development of immune responses, since they can be the initiator of a functional crosstalk that modulates their physiology and homeostatic balance (20). Even though many lectins have been purified and used as bioactive compounds (21), there is no study so far in the literature characterizing proteins from *B. pilosa* with lectin activity.

Considering the effects of *B. pilosa* to control certain diseases and the fact that this plant species has never been studied before to control *T. gondii* infection, the major aim of the present study was to evaluate whether the total extract and an acetonic fraction from this plant could have any effect to control *T. gondii* infection. To achieve this aim, it was designed experiments by using *in vitro* and *in vivo* models.

## Materials and Methods

### Plant Samples

*Bidens pilosa* L. was collected in an experimental area from Institute of Agricultural Sciences (ICIAG), Federal University of Uberlândia (Minas Gerais), Brazil, and identified by Dr. Jimi Naoki Nakajima. A voucher specimen is deposited at the Herbarium II Uberlandensis—HUFU, Institute of Biology, Uberlândia, with accession number 33516.

### Preparation of Total Extract and Acetonic Fraction From *B. pilosa*

Two methods were used to prepare extracts from the air-dried plant samples: (i) total extract was obtained from 10 g samples of the *B. pilosa* whole plant. This material was dissolved in 1,000 mL of boiling distilled water, lightly stirred and covered with gauze for 10 min, recovered by filtration, and the mixture was cooled to room temperature, as described (22, 23); (ii) acetonic fraction of *B. pilosa* was obtained by precipitation process of the total extract by adding cold acetone (1:1) and incubated for 30 min at −70°C, followed by 15 min at −20°C. After centrifugation at 14,000 × g for 20 min at 4°C, the supernatant was discarded and the precipitate was solubilized in 0.9% NaCl. The material was placed in dialysis membranes (cut off 12 kDa), and dialyzed against 0.9% NaCl. Protein concentrations from both preparations were determined by Bradford method (24).

### One-Dimensional (1-DE) and Two-Dimensional (2-DE) Gel Electrophoresis

For 1-DE gel electrophoresis, samples of total extract and acetonic fraction of *B. pilosa* were assessed by 12% polyacrylamide gel electrophoresis under denaturing conditions (SDS-PAGE) in non-reducing conditions (25), in parallel with molecular weight markers (BenchMarkTM Protein Ladder 6–200 kDa, Invitrogen, Karlsruhe, Germany). Gels were stained with Coomassie brilliant blue G-250® (Sigma-Aldrich, St. Louis, MO, USA). The molecular weight bands were estimated by linear regression analysis, based on the calculation of relative mobility (Rf), using KODAK 1D Image Analysis program (Eastman Kodak Co., Rochester, USA). Additionally, the band was submitted to MS/MS and identified in the National Center for Biotechnology Information (NCBI) database.

For 2-DE gel electrophoresis, 60 μg of acetonic fraction were diluted in ultrapure water and separated by isoelectric focusing (IEF) on 7-cm immobilized pH gradient strips (ReadyStripTM IPG Strip pH 3–10) overnight at room temperature, following the manufacturer instructions (GE, Healthcare, Uppsala, Sweden). After IEF, strips were equilibrated and running onto precast 12% polyacrylamide gels, being the staining spots analyzed by ImageMasterTM 2-D Platinum 7.0 (GE Healthcare, Amersham Pharmacia Biotech, United Kingdom) to be submitted to MS/MS and identified in the NCBI database.

### Mass Spectrometry and Analysis *in silico*

The identification of protein present in acetonic fraction from *B. pilosa* was carried out by mass spectrometry, as described previously (26). Briefly, the spot of interest was selected and excised manually from previously stained 2-DE gels. Gel piece was treated with trypsin and the digest was concentrated, desalted, and fractioned by reverse phase chromatography. The peptide was separated, ionized, fragmented and analyzed according to their mass/charge (m/z). The spectra were analyzed by ProteinLynx Global Server (PLGS) 4.2 (Waters, Mildford, MA, USA) and searched in the NCBI database. All MS/MS spectra were analyzed using the Masslynx V 4.1 software (Micromass, UK). The identification of peptide was considered reliable if the sequence coverage exceeded 80%. Subsequently, the sequences of peptides were examined at GenBank and NCBI databases.

### Hemagglutination Assay

Semi-quantitative hemagglutination tests were performed in microtiter plates of 96-well V-bottom, according protocol described by Nowotny (27). It was added in each well 50 μL of the serial diluted fractions plus 25 μL of the 2% erythrocyte suspensions. The control of the reaction consisted of 0.9% NaCl solution. The presence of hemagglutination was examined macroscopically, after incubation for 1 h at room temperature.

### Inhibition of Hemagglutinating Activity

Carbohydrate specificity tests were performed using the following sugars: D(+)-Galactose, D(+)-Glucose, D(+)-Mannose, sucrose, lactose, and D-fructose, in the initial concentrations of 0.2 M following serial dilutions. After adding 5 μL of the acetonic fraction (0.02 μg/well) and incubation for 1 h at room temperature, it was added 25 μL of human erythrocyte suspension 2%, and, after additional 2 h incubation at room temperature, the inhibition of hemagglutinating activity was assessed.

### Cell Culture and Cytotoxicity Assays

HeLa cells, obtained from American Type Culture Collection (ATCC, Manassas, VA, USA), or peritoneal cells, obtained from C57BL/6 mice, were cultured in RPMI-1640 medium supplemented with 25 mM HEPES, 2 mM L-glutamine, 100 U/mL penicillin, 100 μg/mL streptomycin (all reagents from Sigma-Aldrich), and 10% heat-inactivated fetal calf serum (FCS) (Cultilab, Campinas, Brazil) in a humidified incubator at 37°C and 5% CO_2_. The cytotoxicity of the *B. pilosa* preparations was evaluated by MTT assay (28). The cells were cultured in 96 well plate in the presence of two-fold serial dilutions of total extract (5,000–9.8 μg/mL) or acetonic fraction (100–0.39 μg/mL). As controls, cells were incubated with medium alone. After 24 h or 48 h-incubation, cells were washed and incubated for 4 h with 0.5 mg/mL thiazolyl blue tetrazolium (MTT, Sigma-Aldrich). Formazan particles were solubilized in 10% sodium dodecyl sulfate (SDS) and 50% N,N-dimethyl formamide. Absorbance was measured at 570 nm by plate reader (M2e, Molecular Devices) and the results were expressed as percentage of viable cells, compared to controls.

### Parasites

Tachyzoites from 2F1 clone of *T. gondii* RH strain, constitutively expressing cytoplasmic β-galactosidase), were maintained by serial passages in HeLa cells in RPMI 1640 medium supplemented with 2 mM glutamine, 100 U/mL penicillin, 100 μg/mL streptomycin, and 2% heat-inactivated calf fetal serum (FCS) at 37°C in a 5% CO_2_.

Cysts from ME49 strain of *T. gondii* were obtained from brain tissues from C57BL/6 infected 30 days earlier by oral route and prepared as previously described (2). Brains were removed, washed in 0.01 M phosphate-buffered saline (PBS) pH 7.2, homogenized and cysts were counted under light microscopy.

### *In vitro* Assay for *T. gondii* Infection

In a first set of experiments, 2F1 RH tachyzoites were pretreated for 1 h at 37°C and 5% CO_2_ with two-fold serial dilutions of total extract (312.5–9.8 μg/mL) and acetonic fraction (100–3.12 μg/mL) of *B. pilosa* or medium alone. The parasite suspensions containing at least 90% of viable tachyzoites, as determined by Trypan blue staining, were used to infect HeLa cells at 0.5:1 ratio (parasite:cell) to parasite replication for 24 h at 37°C or 1:1 for 1 h at 37°C to infection index measurements. Parasite replication was determined by β-galactosidase colorimetric assay in a plate reader (M2e, Molecular Devices) at 570 nm. The results were expressed as number of tachyzoites in relation to a reference curve of 2F1 RH tachyzoites ranging from 15.6 × 10^3^ to 1 × 10^6^ parasites.

In a second set of experiments, HeLa monolayers were pretreated for 1 h at 37°C and 5% CO_2_ with two-fold serial dilutions of total extract or acetonic fraction from *B. pilosa* or medium alone. Cells were infected with 2F1 RH tachyzoites at 0.5:1 ratio or 1:1, as described above. Parasite replication was determined as described above. Two independent experiments were performed with four replicates for each experimental condition. Results were expressed as percentages of inhibition of infection, as well as of parasite replication for each treatment in relation to controls. The median inhibitory concentration (IC_50_) of each drug was calculated by extrapolation of the corresponding dose–response curve on a log-linear plot employing the portions of the curve that transected the 50% response point (29).

### Animals

Male BALB/c and C57BL/6 mice at 6–10 weeks of age were kept in the Center for Bioterism and Animal Experimentation, Federal University of Uberlandia, Brazil. All animals were maintained in individual cages, under standard laboratory conditions (12 h light and 12 h dark cycle, controlled temperature of 22 ± 2°C), and received food and water *ad libitum*. All procedures were conducted according to the institutional guidelines for animal ethics, as well as to the National Institutes of Health guidelines for the human use of laboratory animals. This study was approved by the Ethics Committee for Animal Experimentation from the Federal University of Uberlândia (CEUA-UFU), under Protocol CEUA/UFU No. 054/11.

### Bioassay to Assess *in vivo* Infectivity of *T. gondii* Tachyzoites After Exposure to *B. pilosa* Extracts

BALB/c mice were infected intraperitoneally by 10^6^ 2F1 RH tachyzoites of *T. gondii* in 0.2 mL, according to the following groups, containing 5 animals per group: (i) tachyzoites pretreated with total extract of *B. pilosa* (300 μg/mL), (ii) tachyzoites pretreated with acetonic fraction of *B. pilosa* (100 μg/mL), and (iii) tachyzoites treated with medium alone (control). The numbers of viable tachyzoites were determined by Trypan blue staining and used to infect the mice. After 3 days of infection, the animals were euthanized and their intraperitoneal cavity washed with 5 mL of PBS and saved for counting parasites by two independent observers by using Trypan blue. In addition, the quantification of the number of parasites was also carried out in parallel by colorimetric analysis of parasites expressing β-galactosidase. The parasite suspensions were added to 96-well plates, centrifuged at 250 × g for 5 min, and lysis buffer was added in volumes for 15 min, followed by addition of assay buffer containing 3 mM CPRG. After 30 min, the optical density was determined at 570 nm by plate reader spectrophotometer (M2e, Molecular Devices).

### *In vivo* Assay for *T. gondii* Infection During Chronic Phase

C57BL/6 mice were infected intraperitoneally by 20 Me49 cysts of *T. gondii*, according to the following groups: (i) mice treated for 10 days intraperitoneally in the same day of the infection with total extract of *B. pilosa* (10 mg/kg), (ii) mice treated with acetonic fraction of *B. pilosa* (2 mg/kg), (iii) mice treated oral via with sulfadiazine (150 mg/kg), and (iv) mice untreated. After, 30 days of infection the serum was collected to determinate of *T. gondii*-specific total IgG, IgG1, and IgG2a antibodies, and the brain to assess the parasite burden.

### Determination of *T. gondii*-Specific Total IgG, IgG1, and IgG2a Antibodies

Levels of *T. gondii*-specific total IgG, IgG1, and IgG2a antibodies were measured by ELISA. High-affinity microtiter plates were coated with Stag (10 μg/ml), washed with PBS plus 0.05% Tween 20 (PBS-T) and blocked with 5% skim milk in PBS-T for 1 h at room temperature. Serum samples were diluted 1:25 in 1% skim milk-PBS-T and incubated for 1 h (for IgG detection) or 2 h (for IgG1 and IgG2a detection) at 37°C. After washing, peroxidase-labeled goat anti-mouse IgG (1:1,000; Sigma Chemical Co., St Louis, MO) or biotin-labeled goat anti-mouse IgG1 (1:4,000) or anti-mouse IgG2a (1:2,000) antibodies (Caltag Lab. Inc., South San Francisco, CA) were added and incubated for 1 h at 37°C. Next, streptavidin-peroxidase (1:1,000; Sigma) was added for IgG1 and IgG2a detection assays. The assays were developed with 0.01 M 2,2-azino-bis-3-ethyl-benzthiazoline sulfonic acid (ABTS; Sigma) and 0.03% H_2_O_2_. Optical density (OD) values were determined in a plate reader (M2e, Molecular Devices) at 405 nm. Results were expressed in ELISA index (EI) to the formula: EI = OD sample/OD cut off, where cut off was calculated as the mean OD for negative control sera plus three standard deviations.

### Determination of Parasite Burden Using Real-Time PCR (qPCR)

Brain parasite load was determined by quantitative real time PCR using primer pairs (sense 3′-GCTCCTCCAGCCGTCTTG-5′; antisense 3′-TCCTCACCCTCGCCTTCAT-5′) to detect the *T. gondii* Tg529 sequence through SYBR green system (GoTaq qPCR Mater Mix, Promega) as previously described (30). DNA extraction was performed from 20 mg of murine brain tissues (Genomic DNA kit, Promega Co., Madison, WI) and parasite loads were calculated by interpolation from a standard curve established by a 10-fold serial dilution of 100 ng of the DNA included in the reaction. All samples were standardized for 200 ng of DNA and run in triplicate with the negative controls.

### Statistical Analysis

Statistical analysis was carried out using GraphPad Prism version 6.0 (GraphPad Software Inc., San Diego, USA). After passed to the normality tests, values were expressed as mean ± standard deviation, and analyzed by one-way ANOVA test, followed by Bonferroni *post hoc* test for comparison among the groups, considering *P* < 0.05 as significant.

## Results

### Identification of Maturase K in Acetonic Fraction From *B. pilosa*

**Figure 1:**
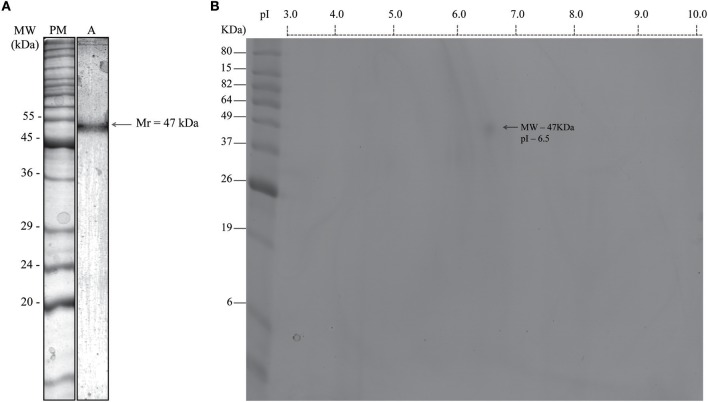
Identification of maturase k in acetonic fraction from *Bidens pilosa* stained by Coomassie brilliant blue G-250®, resolved by 12% SDS-PAGE. **(A)** One-dimensional gel electrophoresis (1-DE). **(A)** Apparent molecular mass (Mr) of the *B. pilosa* acetonic fraction is shown on the right as determined by Kodak 1D image analysis. Representative gel image from four independent experiments. **(B)** Two-dimensional gel electrophoresis (2-DE). Isoelectric focusing (IEF) was performed on 7 cm immobilized pH gradient strips (ReadyStrip^TM^ IPG Strip pH 3–10). The protein spot was determined by ImageMasterTM 2D Platinum 7.0 software. The spot is identified by arrow. Molecular weight markers (MW) are indicated on the left in kiloDalton (kDa) and the range of isoelectric point (pI) is indicated above the panel. Representative gel image from two matched independent experiments.

The total extract of *B. pilosa* was tested at different concentrations from a stock solution (10 mg/mL), based on the proportion of dry weight per volume of water for infusion. The acetonic fraction obtained from the total extract showed protein concentration varying from 150 to 450 μg/mL in different preparations. The single band was detected in acetonic fraction of *B. pilosa* corresponded to apparent molecular weight (MW) of 47 kDa (Figure 1A) by one-dimensional gel electrophoresis (1-DE). Also, this fraction showed only one spot obtained with pI of 6.5 and Mr of 47 kDa (Figure 1B) by two-dimensional gel electrophoresis (2-DE) and, based on the results of protein identification and the amino acid sequence by mass spectrometry (MS), it was determined that this component is maturase K (Supplementary Table 1S). The single band was also detected in other conditions when both extracts of *B. pilosa* in 1-DE was silver stain (Supplementary Figure 1SA) and native gel Coomassie stain (Supplementary Figure 1SB). However, when stain with periodic acid-Schiff (PAS) was not detected any band (Supplementary Figure 1SC).

### Maturase K Is a Lectin That Can Be Isolated by Three Carbohydrates

**Figure 2:**
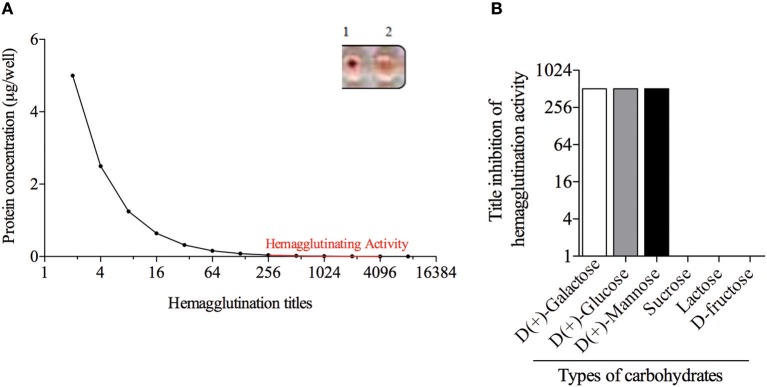
*Bidens pilosa* acetonic fraction has hemagglutination activity. **(A)** Dilutions of *Bidens pilosa* protein was added to human erythrocytes suspensions. Aspect of the hemagglutination assay showing negative reaction (1) and positive reaction (2). Representative results from two independent experiments. **(B)** Inhibition of hemagglutination activity of *Bidens pilosa* acetonic fraction by six carbohydrates: [D(+)-Galactose, D(+)-Glucose, D(+)-Mannose, sucrose, lactose, and D-fructose]. Hemagglutination activity inhibition was observed by D(+)-Galactose, D(+)-Glucose and D(+)-Mannose. Representative results from two independent experiments.

The *B. pilosa* preparations showed different titers of hemagglutination on the erythrocytes panel (Figure 2). Results of this assay showed the presence of a *B. pilosa* lectin that agglutinates human erythrocytes of B or AB type, with titers from 256 to 4,096 (Figure 2A), compared with negative reactions. The inhibitory action of carbohydrates on the hemagglutinating activity of the acetonic fraction was analyzed against six different sugars, but only three of them [D(+)-Galactose, D(+)-Glucose and D(+)-Mannose] showed inhibitory activity (Figure 2B). These results showed the lectinic effect of the protein isolated in the *B. pilosa* acetonic fraction that binging in the D(+)-Galactose, D(+)-Glucose and D(+)-Mannose carbohydrates.

### Total Extract and Acetonic Fraction From *B. pilosa* Induce Low Cytotoxicity for Host Cells

**Figure 3:**
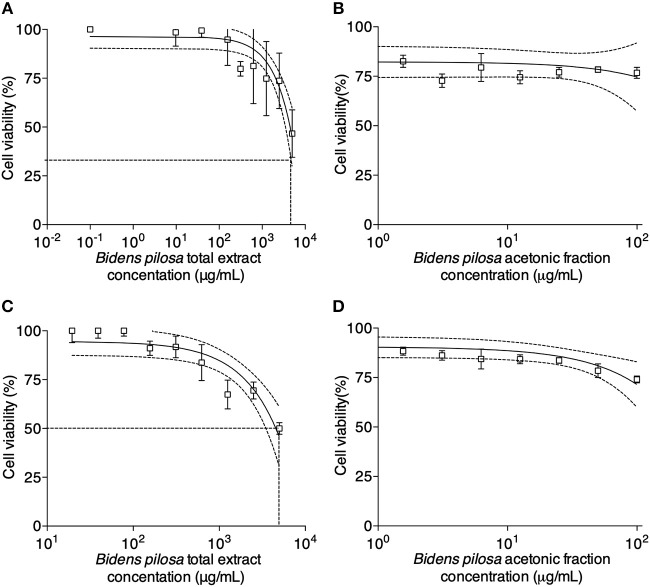
*In vitro* cytotoxicity activity determined by MTT assay for HeLa and peritoneal cells from C57BL/6 mice. HeLa cells were cultured in 96-well plates in the absence (control) or presence of different concentrations of **(A)** *Bidens pilosa* total extract (from 5,000 to 9.8 μg/mL) or **(B)** *Bidens pilosa* acetonic fraction (from 100 to 0.8 μg/mL) for 24 h. Results are expressed as percentage of viable cells compared to control. Peritoneal cells were cultured in 96-well plates in the absence (control) or presence of different concentrations of **(C)** *Bidens pilosa* total extract (from 5,000 to 9.8 μg/mL) or **(D)** *Bidens pilosa* acetonic fraction (from 100 to 0.8 μg/mL) for 48 h. Results are expressed as percentage of viable cells compared to control. Values are representative from three independent experiments, indicating mean ± SD.

Cell viability was determined for different concentrations of *B. pilosa* preparations, using serial two-fold dilutions ranging from 5,000 to 9.8 μg/mL (total extract) and 100 to 0.8 μg/mL (acetonic fraction). It was observed that cell viability rates ranged from 47 to 77% in the highest concentrations of total extract (Figure 3A) or acetonic fraction (Figure 3B) in HeLa cells for 24 h, respectively and from 50 to 74% in the highest concentrations of total extract (Figure 3C) or acetonic fraction (Figure 3D) in peritoneal cells from C57BL/6 mice for 48 h, respectively. The median cytotoxic doses (toxic dose 50%–DT_50_) for total extract and acetonic fraction of *B. pilosa* were calculated in a dose-response curve corresponding to a log-linear graph, obtaining a correlation coefficient of 0.960 and DT_50_ of 6563.0 μg/mL for total extract (Figure 3A) and correlation coefficient of 0.958 and DT_50_ of 288.5 μg/mL for acetonic fraction (Figure 3B).

### Total Extract and Acetonic Fraction From *B. pilosa* Decrease *in vitro T. gondii* Infection

**Figure 4:**
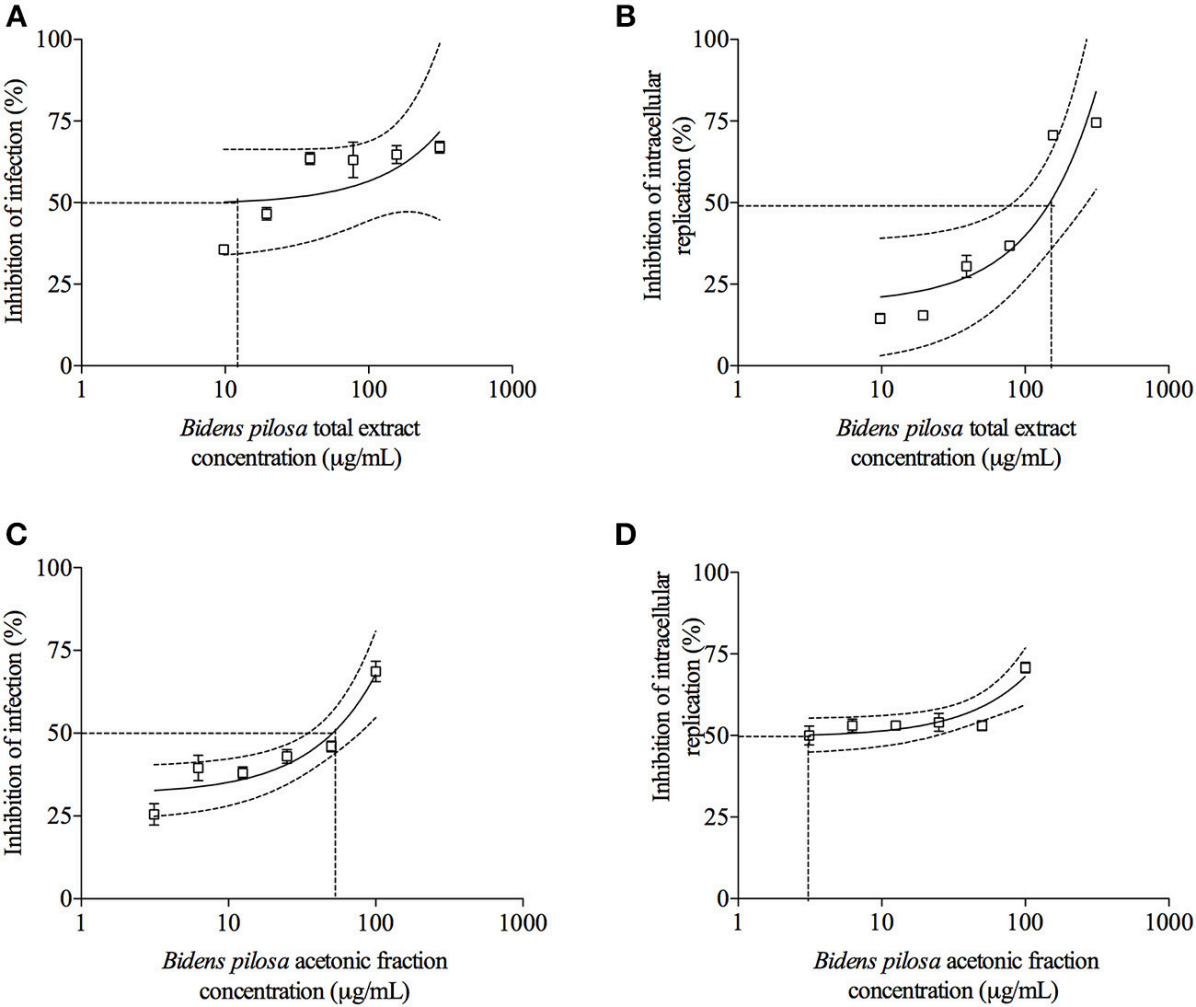
Effects of treatment with total extract or acetonic fraction of *Bidens pilosa* on *Toxoplasma gondii* infection index and intracellular replication in HeLa cells. Treatments were carried out on *T. gondii* tachyzoites before infection of HeLa cells with total extract **(A,B)** or acetonic fraction **(C,D)**. Results are expressed as inhibition of percentage of infection **(A,C)** and parasite intracellular replication **(B,D)** in comparison to controls. Dotted lines show the inhibitory concentration of 50% (IC_50_) and bars represent standard deviations. Values are representative from three independent experiments, indicating mean ± SD.

**Figure 5:**
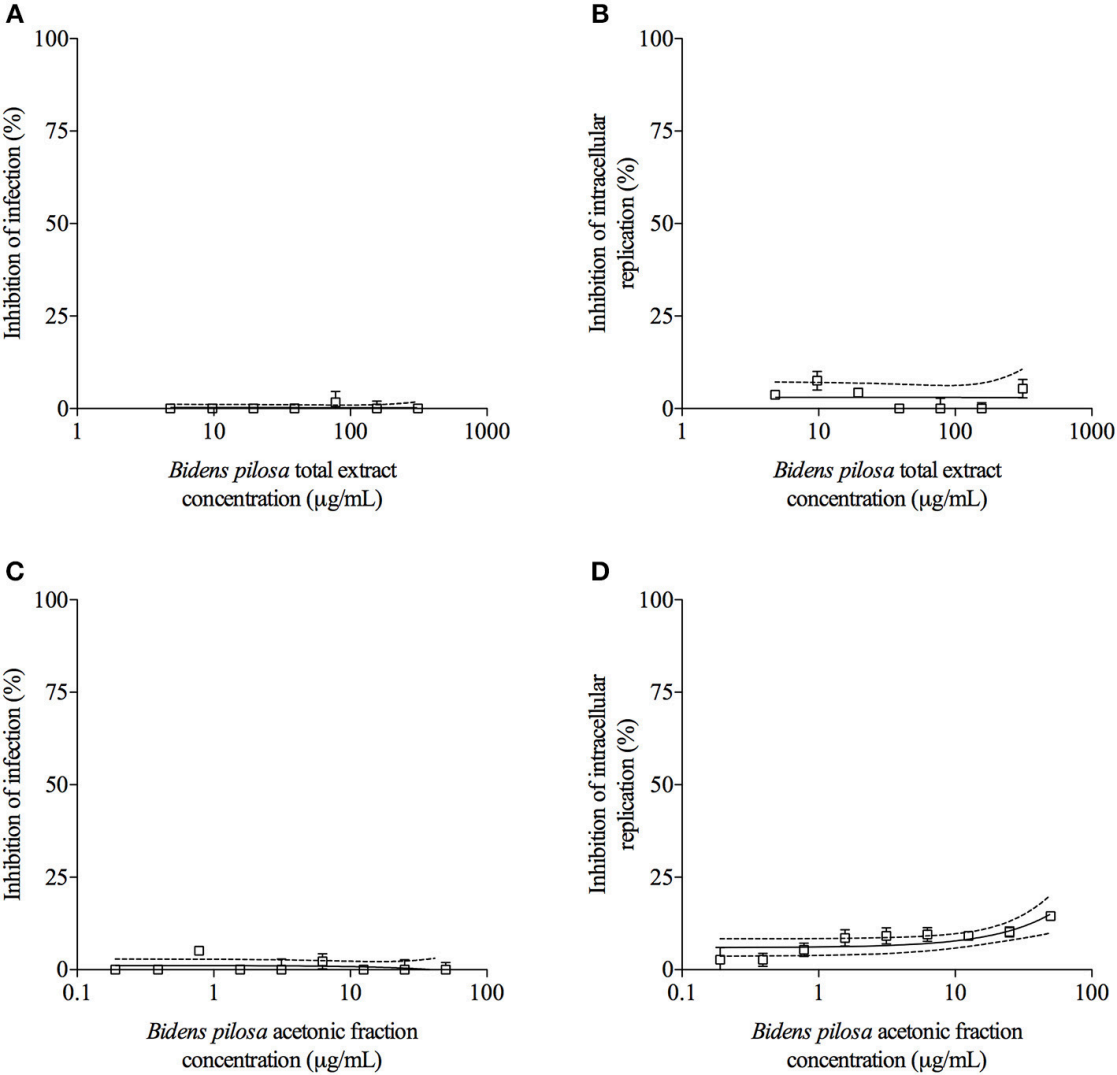
Effects of treatment with *B. pilosa* on HeLa cells in *T. gondii* infection index and intracellular replication of the parasite. Treatments were carried out before *T. gondii* infection on HeLa cells with total extract **(A,B)** or acetonic fraction **(C,D)** of *B. pilosa*. Results are expressed as inhibition of percentage of infection **(A,C)** and parasite intracellular replication **(B,D)** in comparison to controls. Dotted lines show the inhibitory concentration of 50% (IC_50_) and bars represent standard deviations. Values are representative from three independent experiments, indicating mean ± SD.

**Figure 6:**
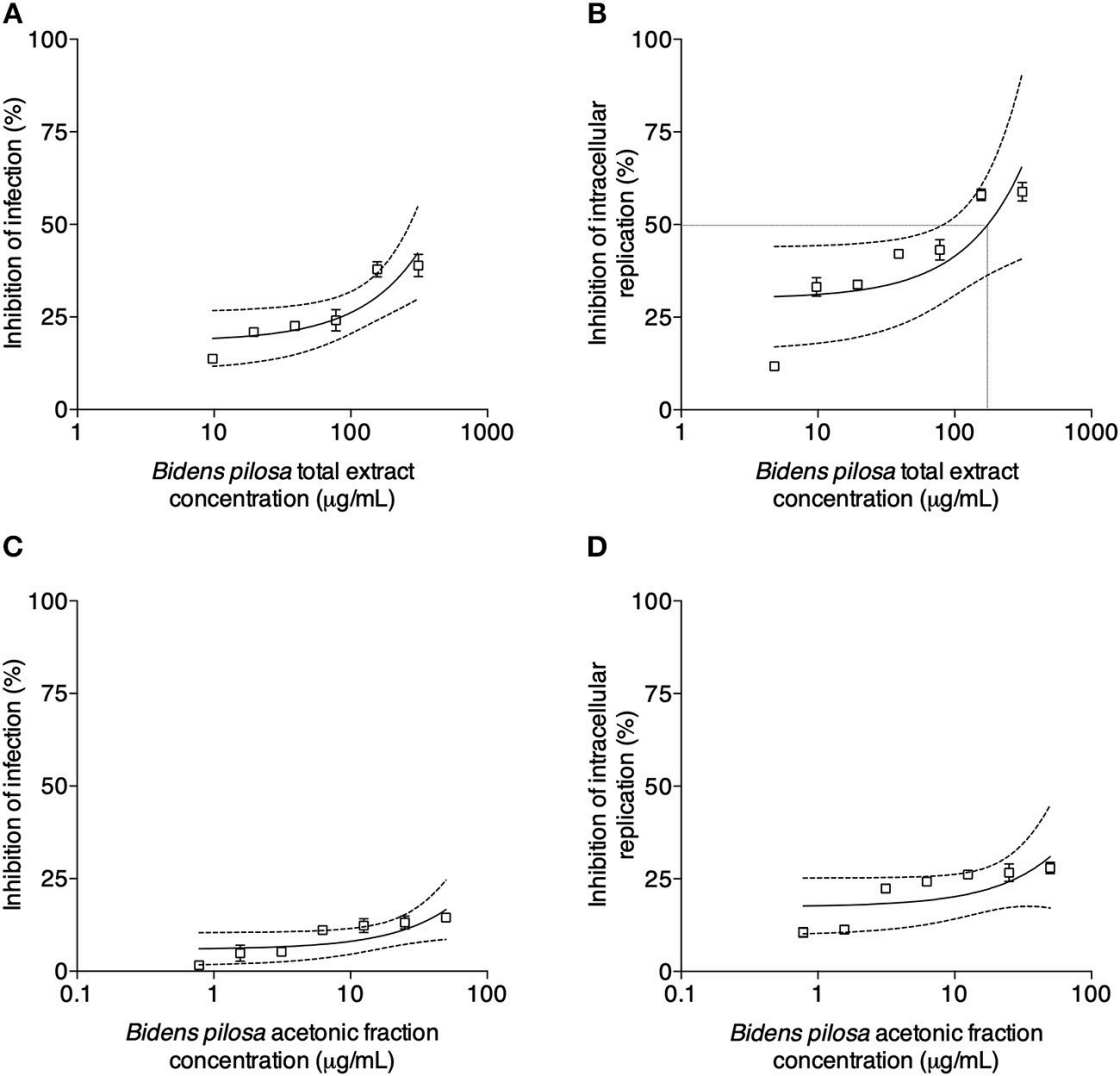
Effects of treatment with *B. pilosa* on HeLa cells infected by *T. gondii*. Treatments were carried out after *T. gondii* infection on HeLa cells with total extract **(A,B)** or acetonic fraction **(C,D)** of *B. pilosa*. Results are expressed as inhibition of percentage of infection **(A,C)** and parasite intracellular replication **(B,D)** in comparison to controls. Dotted lines show the inhibitory concentration of 50% (IC_50_) and bars represent standard deviations. Values are representative from three independent experiments, indicating mean ± SD.

*T. gondii* tachyzoites were pre-treated with different concentrations of extracts of *B. pilosa*, it was observed that parasites receiving the highest concentration of total extract or acetonic fraction showed a significant decrease for both parameters analyzed, i.e., infection and parasite replication. The rates of infection and replication inhibition of the parasites were 67 and 74%, respectively, when they were pre-treated with the total extract (312.5 μg/mL) (Figures 4A,B), and 68 and 70%, respectively, when pre-treated with the acetonic fraction (100 μg/mL), compared to the control group (Figures 4C,D). In contrast, the pretreatment of HeLa cells with different concentrations of total extract or acetonic fraction of *B. pilosa*, which were washed out before infection by *T. gondii* tachyzoites, has no effect on infection, as it was not observed a significant inhibition of infection and parasite replication at any concentration of both extracts of the plant when compared to controls (Figure 5). The inhibition rate of infection was extremely low (below 5%) and replication of the parasite was 5.5% for the total extract (Figures 5A,B) and 14% for the acetonic fraction, using the highest concentration of the both preparations (Figures 5C,D). There was no significant difference when comparing the rates of infection and replication of the parasite among pre-treated cells. In another condition, the cells were infected with *T. gondii* tachyzoites, and afterwards treated with different concentrations of total extract or acetonic fraction of *B. pilosa*. When infected cells were treated, it was observed that cell cultures receiving the highest concentration of total extract or acetonic fraction showed decreases for both parameters analyzed. The rates of infection and replication inhibition of the parasites were 37 and 58%, respectively, when they were treated with the total extract (312.5 μg/mL) (Figures 6A,B), and 15 and 28%, respectively, when treated with the acetonic fraction (100 μg/mL), compared to the control group (Figures 6C,D). As shown in Table 1, the toxic dose 50% (DT_50_), the inhibitory dose of 50% (DI_50_) and the corresponding therapeutic index (TI) for both extracts when the three different conditions of treatment were calculated, based on the results obtained from cytotoxicity experiments, the inhibition rates of infection, and parasite replication. When *T. gondii* tachyzoites were treated with the extracts prior of infection of HeLa cells, the total extract showed ID_50_ of 21.0 μg/mL in infection and 92.2 μg/mL in replication, with DT_50_ of 6563.0 μg/mL. For treatment with acetonic fraction, the ID_50_ values were 52.0 μg/mL in infection and 3.1 μg/mL in replication, with DT_50_ of 288.5 μg/mL. Consequently, the calculated TI for total extract of *B. pilosa* in infection (TI = 312.5) was higher than that found for acetonic fraction (TI = 5.6). Regarding the parasite replication, TI for acetonic fraction (TI = 92.5) was higher than for total extract (TI = 71.3). Also, TI could not be calculated in the treatment condition of HeLa cells prior of infection with tachyzoites of *T. gondii* and in the cells infected and treated, since the inhibitory dose of 50% could not be calculated, except for the replication in the cells infected treated with total extract, with ID_50_ of 181.6 μg/mL (TI = 134.5). Thus, total extract and acetonic fraction decreased the invasion and replication of *T. gondii* when the parasites were pre-treated directly, whereas the parasite replication decreased when cells were infected and treated afterwards with the total extract.

**Table 1:** Effects of *Bidens pilosa* total extract or acetonic fraction treatments on infection and intracellular replication of *Toxoplasma gondii* in HeLa cells under different conditions.

**Pretreatment**	**Drugs**	${\text{DT}}_{50}^{\text{d}}$ **(μg/mL)**	${\text{ID}}_{50}^{\text{e}}$ **(μg/mL)**		**TI**^**f**^	
			**Infection**	**Replication**	**Infection**	**Replication**
Parasites^a^	Total extract of *B. pilosa*	6563.0	21.0	92.2	312.5	71.3
	Acetonic fraction of *B. pilosa*	288.5	52.0	3.1	5.6	92.5
Cells^b^	Total extract of *B. pilosa*	6563.0	ND^g^	ND^g^	ND^g^	ND^g^
	Acetonic fraction of *B. pilosa*	288.5	ND^g^	ND^g^	ND^g^	ND^g^
Infected cells^c^	Total extract of *B. pilosa*	6563.0	ND^g^	181.6	ND^g^	134.5
	Acetonic fraction of *B. pilosa*	288.5	ND^g^	ND^g^	ND^g^	ND^g^

a *Treatment of T. gondii tachyzoites with B. pilosa prior to infection of HeLa cells*.

b *Treatment of HeLa cells with B. pilosa prior to infection with T. gondii tachyzoites*.

c *Infection of HeLa cells with T. gondii tachyzoites prior to treatment with B. pilosa*.

d *DT_50_, toxic dose of 50% in HeLa*.

e *ID_50_, inhibitory dose of 50%*.

f *TI, therapeutic index = DT_50_/ID_50_*.

g *Inhibition of 50% could not be determined %*.

### Total Extract and Acetonic Fraction From *B. pilosa* Decrease *in vivo* Acute *T. gondii* Infection

**Figure 7:**
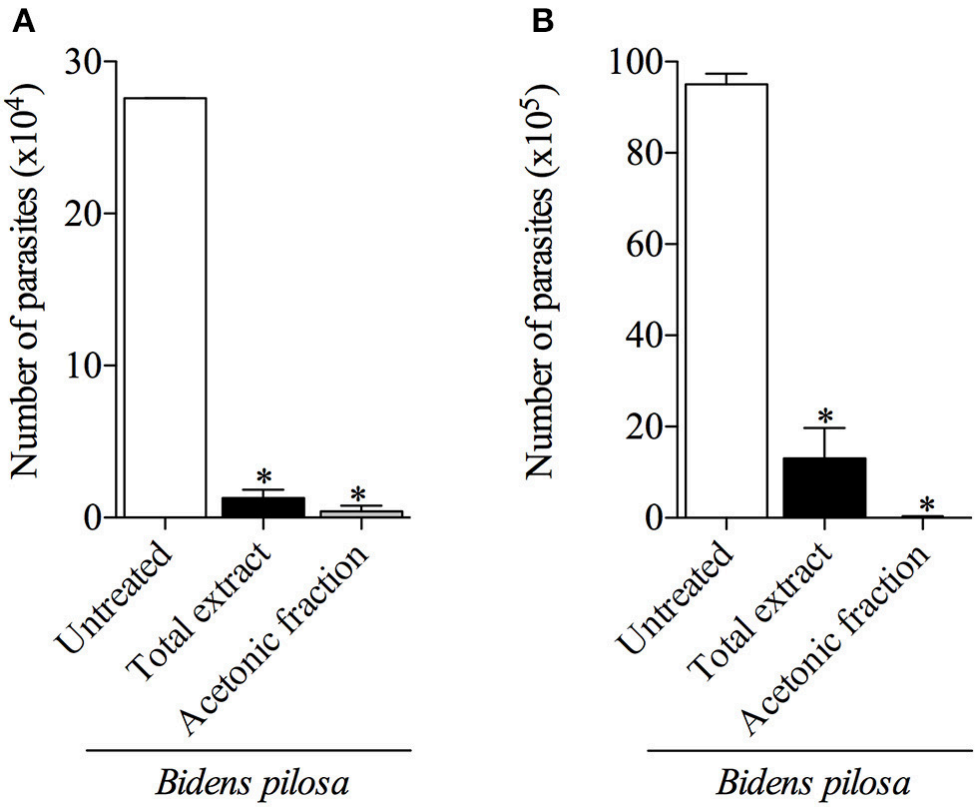
Effects of pretreatment of *Toxoplasma gondii* tachyzoites with total extract or acetonic fraction of *Bidens pilosa* in BALB/c mice infection. The peritoneal exudates from mice were obtained after 3 days of infection and the numbers of parasites were assessed by colorimetric reaction **(A)** or in Neubauer chamber **(B)**. Values are indicated as mean ± SD of parasites in the peritoneal exudate in relation to untreated parasites. ^*^indicate statistically significant differences for untreated (ANOVA and Bonferroni multiple comparison *post hoc* test; *P* < 0.05).

When parasites were treated with 300 μg/mL of total extract or 100 μg/mL of acetonic fraction of *B. pilosa* and used to infect mice, it was observed a significant decrease in the number of both extra- and intra-cellular parasites recovered by peritoneal lavage of those animals, when compared with the group of animals infected by untreated parasites (Figures 7A,B). The rates of the parasite inhibition were 99.9%, when treated by acetonic fraction, 91%, when treated with the total extract, as determined by colorimetric reaction, and 99.6 and 86.4%, respectively, when evaluating by Neubauer chamber counting. Hence, both total extract and acetonic fraction of *B. pilosa* were able to reduce infectivity of tachyzoites when pretreated in these preparations.

### Total Extract and Acetonic Fraction From *B. pilosa* Decrease *in vivo* Chronic *T. gondii* Infection

**Figure 8:**
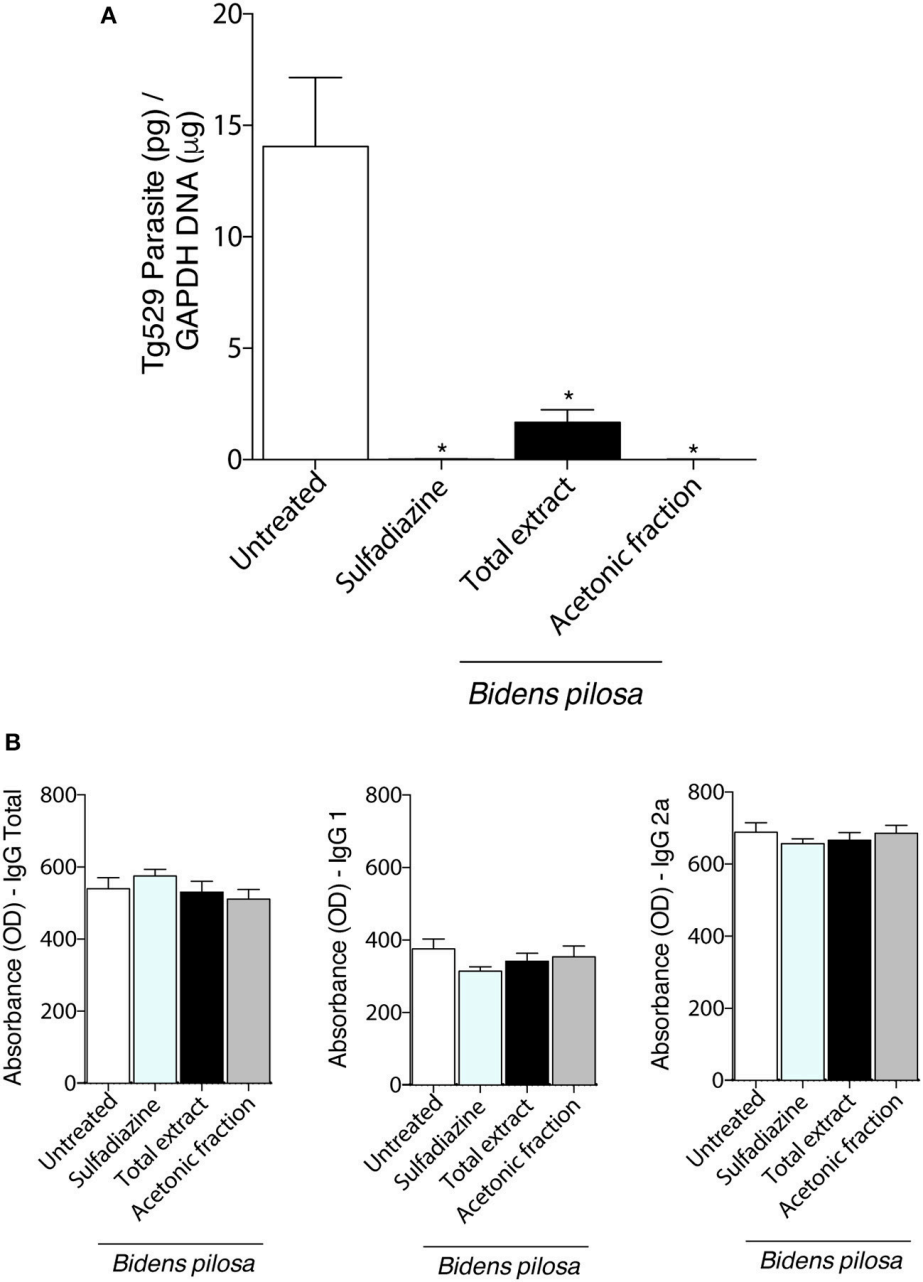
Effects of the treatment with total extract or acetonic fraction of *Bidens pilosa* in C57BL/6 mice infected for 30 days with Me49 strain *Toxoplasma gondii* cysts. Four groups of mice (10 animals per group) were infected and treated with total extract or acetonic fraction of *Bidens pilosa*. As control, mice were treated with PBS only (infection control—Untreated). After treatment, the brain and serum were collected. **(A)** Brain parasite burden in the four groups determined by qPCR. **(B)** Comparison between IgG total, IgG1, and IgG2a responses to *T. gondii*. Values are indicated as mean ± SD of treated mice in relation to untreated ones. ^*^indicate statistically significant differences in comparison with untreated animals (ANOVA and Bonferroni multiple comparison *post hoc* test; *P* < 0.05).

Brain parasite burden in mice chronically infected with cysts of Me49 strain of *T. gondii* determined by real-time PCR (Figure 8A) was lower in mice treated with total extract, acetonic fraction of *B. pilosa* or sulfadiazine groups than untreated group (*P* < 0.05). To confirm that the animals from all groups were really infected, the presence of antibodies in the serum of those mice were also evaluated by ELISA (Figure 8B), showing similar profile among of them, as the levels of total IgG, IgG1, and IgG2a antibodies did not present significant differences, with a lower production of IgG1 and higher IgG2a in infected mice. Thus, the treatment with total extract or acetonic fraction of *B. pilosa* induce significant decrease of the parasite burden in brain tissue of *T. gondii* infected mice.

## Discussion

Toxoplasmosis has relevance in veterinary sciences, considering the significant economic loss due to abortions, particularly in ovine herds, and for particular groups of humans, such as pregnant women and immunocompromised patients, when the consequences of infection are more severe. Since current treatment of toxoplasmosis is challenging, because of the toxic effects of the available drugs, it has been carried out many studies, including those involving the ethnopharmacological aspects, trying to generate new and more effective drugs, which can act successfully on the parasite, but avoiding the severe side effects to the hosts observed with the currently available drugs (31, 32).

Chinese literature for millennia has been describing the use of *B. pilosa* for treatment of diabetes, bacterial infections, tumors, and infections by protozoan parasites, as those caused by *Plasmodium* spp. (12–14, 16, 33). In this context, the present study aimed to assess the effect of *B. pilosa* on *T. gondii* tachyzoites, mainly from an acetonic fraction, presenting a single component from a protein showing lectin activity, which was characterized as maturase K. Many lectins have been purified and used as biotechnological tools and bioactive compounds and the importance to identify and characterize new lectins is clear (21). In the present study, the lectin from *B. pilosa*, identified as maturase K could be purified using D(+)-Galactose, D(+)-Glucose, and D(+)-Mannose.

The maturase k is conserved and present in mitochondria and chloroplasts from plants, fungi, and bacteria. Its precise function is so far unknown, but this protein is involved in the removal of introns from RNA transcripts, making it vital for these organisms (34–36). This molecule has important implications in many biological fields nowadays, including the rapid biodiversity assessment for food chain analysis, monitoring of protected species, early identification of invasive pest species, identification of active compounds, as well as to control pathogens and their vectors (37–39). Apicomplexan parasites, such as *Plasmodium* sp. and *T. gondii*, hold a residual plastid homologous to the chloroplasts of plants, called apicoplast, which is non-photosynthetic, but retains many features of its ancestry, including a circular genome, which is involved in protein synthesis from a group of biosynthetic pathways from cyanobacterial origin (40). However, it is unclear up to now whether apicomplexan parasites present maturase k in their apicoplast, in contrast to many organisms. Thus, based on our findings, it is suggested that maturase K extracted from *B. pilosa* may be acting on *T. gondii* apicoplast, by interfering in its proteins synthesis, considering that parasites pretreated with maturase K showed reduction in infection and replication rates.

In the present study, the cytotoxicity assays revealed that *B. pilosa* whole extract showed more cytotoxicity compared to acetonic fraction, probably because the crude sample could contain more compounds that may be acting in synergism in the extract. Thus, these analyses were important to determine the dosage to be used to maintain high cell viability for the host cells. In addition, both total extract and acetonic fraction of *B. pilosa* showed high efficacy to control the infection and parasite replication, as demonstrated by dose-dependent inhibition curves and considerably low IC_50_ values obtained for each preparation. Moreover, to compare the effectiveness of both *B. pilosa* preparations against the parasite, the therapeutic index was calculated considering not only the inhibitory capacity of infection and replication, but also their toxicity to the host cells. Total extract showed a therapeutic index higher than the acetonic fraction (IT = 312.5 vs. 5.6 μg/mL, respectively) for infection rate. However, considering the replication parameter, both extracts were effective, reflecting the high therapeutic indices obtained, being the acetonic fraction index higher than the total extract one (IT = 92.5 vs. 71.3 μg/mL, respectively). Hence, it may be hypothesized that other components of the total extract may be contributing along with the lectin to a more direct and effective effect to control parasite infection. However, when HeLa cells were pretreated with different concentrations of total extract or acetonic fraction and then infected by *T. gondii*, no significant inhibition was found in the rates of infection or parasite replication, when compared with controls, impeding to calculate the inhibitory dose or the therapeutic index in these conditions.

Regarding the *in vitro* experiments, it was observed that the pretreatment of *T. gondii* tachyzoites before infection of HeLa cells demonstrated a significant effect compared with the treatment of the host cells after *T. gondii* infection and infected cells. These findings suggest that both total extract and acetonic fraction of *B. pilosa* appear to affect more directly the extracellular parasite. Indeed, it was described recently that *B. pilosa* extract has beneficial effects to control coccidiosis in chickens (33). As the *in vitro* results showed significant effect to inhibit infection and replication of the parasite pretreated with *B. pilosa*, it was carried out *in vivo* experiments, by using proper concentrations of total extract or acetonic fraction when compared with the *in vitro* experiments. It was observed that both preparations of *B. pilosa* also played a significant role to inhibit *T. gondii* infectivity under *in vivo* conditions of treatment of the parasite. Interesting, when mice were treated for 10 days after infection, a significant reduction in the parasite burden was observed in the brain tissue of the animals. Overall, these results demonstrate that both total extract and acetonic fraction from *B. pilosa* are able to induce lower parasite infectivity. Therefore, this anti-*Toxoplasma* activity detected for the components from *B. pilosa*, particularly for maturase K lectin, in addition to the absence of cytotoxicity to the host cells, may constitute a useful alternative therapy for toxoplasmosis. In this context, future studies will be necessary to investigate the precise mechanism underlying the inhibition process of the parasite infectivity to the hosts after treatment with maturase K from *B. pilosa*.

## Author Contributions

CM, TM, and JM designed the experiments and analyzed the data. CM, FS, MC, CR, DS, and TdO performed the experiments. CP carried out the mass spectrometry experiments. CM and JM wrote the paper.

### Conflict of Interest Statement

The authors declare that the research was conducted in the absence of any commercial or financial relationships that could be construed as a potential conflict of interest.

## Supplementary Material

Supplementary Figure 1S One-dimensional gel electrophoresis (1-DE), resolved by 12% SDS-PAGE and stained by silver stain **(A)** or native gel **(B)** stained by Coomassie brilliant blue G-250® or periodic acid-Schiff—PAS **(C)**. MW, molecular weight markers; 1, total extract of *B. pilosa*; 2, acetonic fraction of *B. pilosa*. Arrow—component identified as maturase k in the acetonic fraction from *Bidens pilosa*.

Supplementary Table 1S Analyses of Mass Spectrometry (MS) of *Bidens pilosa* acetonic fraction from two-dimensional and one-dimensional gel by NCBI Database (https://www.ncbi.nlm.nih.gov).

## References

[1] Caldas L de Souza W. A Window to *Toxoplasma gondii* Egress. Pathogens. (2018) 7:69. 10.3390/pathogens7030069 30110938 PMC6161258

[2] Berger-Schoch AE Herrmann DC Schares G Müller N Bernet D Gottstein B. Prevalence and genotypes of *Toxoplasma gondii* in feline faeces (oocysts) and meat from sheep, cattle and pigs in Switzerland. Vet Parasitol. (2011) 177:290–7. 10.1016/j.vetpar.2010.11.046 21183278

[3] Mirza Alizadeh A Jazaeri S Shemshadi B Hashempour-Baltork F Sarlak Z Pilevar Z. A review on inactivation methods of *Toxoplasma gondii* in foods. Pathog Glob Health. (2018) 112:306–19. 10.1080/20477724.2018.1514137 30346249 PMC6381540

[4] Pifer R Yarovinsky F. Innate responses to *Toxoplasma gondii* in mice and humans. Trends Parasitol. (2011) 27:388–393. 10.1016/j.pt.2011.03.009 21550851 PMC3159709

[5] Yarovinsky F. Innate immunity to *Toxoplasma gondii* infection. Nat Rev Immunol. (2014) 14:109–21. 10.1038/nri3598 24457485

[6] Skariah S Mcintyre MK Mordue DG. *Toxoplasma gondii*: determinants of tachyzoite to bradyzoite conversion. Parasitol Res. (2010) 107:253–60. 10.1007/s00436-010-1899-6 20514494 PMC3327608

[7] Foroutan M Zaki L Ghaffarifar F Recent progress in microneme-based vaccines development against *Toxoplasma gondii*. Clin Exp Vaccine Res. (2018) 2:93–103. 10.7774/cevr.2018.7.2.93 PMC6082678 30112348

[8] Alday PH Doggett JS. Drugs in development for toxoplasmosis: advances, challenges, and current status. Drug Des Devel Ther. (2017) 11:273–93. 10.2147/DDDT.S60973 28182168 PMC5279849

[9] Paquet C Yudin MH. Toxoplasmosis in pregnancy: prevention, screening, and treatment. J Obstet Gynaecol Can. (2013) 35:78–90. 10.1016/j.jogc.2018.05.036 23343802

[10] Eissa MM El-Azzouni MZ Mady RF Fathy FM Baddour NM. Initial characterization of an autoclaved *Toxoplasma* vaccine in mice. Exp Parasitol. (2012) 131:310–6. 10.1016/j.exppara.2012.05.001 22595548

[11] Zhou Y Fomovska A Muench S Lai BS Mui E McLeod R. Spiroindolone that inhibits PfATPase4 is a potent, cidal inhibitor of *Toxoplasma gondii* tachyzoites *in vitro* and *in vivo*. Antimicrob Agents Chemother. (2014) 58:1789–92. 10.1128/AAC.02225-13 24366743 PMC3957861

[12] Chiang YM Chang CL Chang SL Yang WC Shyur LF. Cytopiloyne, a novel polyacetylenic glucoside from *Bidens pilosa*, functions as a T helper cell modulator. J Ethnopharmacol. (2007) 110:532–8. 10.1016/j.jep.2006.10.007 17101254

[13] Tobinaga S Sharma MK Aalbersberg WG Watanabe K Iguchi K Narui K. Isolation and identification of a potent antimalarial and antibacterial polyacetylene from *Bidens pilosa*. Planta Med. (2009) 75:624–8. 10.1055/s-0029-1185377 19263339

[14] Chien SC Young PH Hsu YJ Chen CH Tien YJ Shiu SY. Anti-diabetic properties of three common *Bidens pilosa* variants in Taiwan. Phytochemistry. (2009) 70:1246–54. 10.1016/j.phytochem.2009.07.011 19683775

[15] Leandro Peixoto Ferreira de Souza Ramos EL Santana SS Silva MV Santiago FM Mineo TW Lectins from *Synadenium carinatum* (ScLL) and *Artocarpus heterophyllus* (ArtinM) are able to induce beneficial immunomodulatory effects in a murine model for treatment of *Toxoplasma gondii* infection. Front Cell Infect Microbiol. (2016) 6:164 10.3389/fcimb.2016.00164 27933277 PMC5122570

[16] Wang R Wu QX Shi YP. Polyacetylenes and flavonoids from the aerial parts of *Bidens pilosa*. Planta Med. (2010) 76:893–6. 10.1055/s-0029-1240814 20108176

[17] Fotso AF Longo F Djomeni PD Kouam SF Spiteller M Dongmo AB. Analgesic and antiinflammatory activities of the ethyl acetate fraction of *Bidens pilosa* (Asteraceae). Inflammopharmacology. (2014) 22:105–14. 10.1007/s10787-013-0196-2 24242914

[18] Le J Lu W Xiong X Wu Z Chen W Anti-Inflammatory Constituents from *Bidens frondosa*. Molecules. (2015) 10:18496–510. 10.3390/molecules201018496 PMC6332032 26473814

[19] Cardoso MRD Mota CM Ribeiro DP Santiago FM Carvalho JV Araujo ECB. ArtinM, a d-mannose-binding lectin from *Artocarpus integrifolia*, plays a potent adjuvant and immunostimulatory role in immunization against *Neospora caninum*. Vaccine. (2011) 29:9183–93. 10.1016/j.vaccine.2011.09.136 22001880

[20] Vasta GR. Roles of galectins in infection. Nat Rev Microbiol. (2009) 7:424–38. 10.1038/nrmicro2146 19444247 PMC3759161

[21] Peumans WJ Van Damme EJ. Plant lectins: specific tools for the identification, isolation, and characterization of O-linked glycans. Crit Rev Biochem Mol Biol. (1998) 33:209–58. 9766939

[22] Camargo ME Berdeja B Miranda G. Diuretic effect of the aqueous extract of *Bidens odorata* in the rat. J Ethnopharmacol. (2004) 95:363–6. 10.1016/j.jep.2004.08.005 15507361

[23] Hsu YJ Lee TH Chang CL Huang YT Yang WC. Anti-hyperglycemic effects and mechanism of *Bidens pilosa* water extract. J Ethnopharmacol. (2009) 122:379–83. 10.1016/j.jep.2008.12.027 19162158

[24] Bradford MM. A rapid and sensitive method for the quantitation of microgram quantities of protein utilizing the principle of protein-dye binding. Anal Biochem. (1976) 72:248–54. 942051 10.1016/0003-2697(76)90527-3

[25] Laemmli UK. Cleavage of structural proteins during the assembly of the head of bacteriophage T4. Nature. (1970) 227:680–5. 5432063 10.1038/227680a0

[26] Pajuaba AC Silva DA Almeida KC Cunha-Junior JP Pirovani CP Camillo LR. Immunoproteomics of *Brucella abortus* reveals differential antibody profiles between S19-vaccinated and naturally infected cattle. Proteomics. (2012) 12:820–31. 10.1002/pmic.201100185 22539433

[27] Nowotny F. Diagnosis of salmonella at the abattoir in Vienna. Wien Tierarztl Monatsschr. (1969) 56:428–32. 5395698

[28] Mosmann T. Rapid colorimetric assay for cellular growth and survival: application to proliferation and cytotoxicity assays. J Immunol Methods. (1983) 65:55–63. 6606682 10.1016/0022-1759(83)90303-4

[29] Jones-Brando L D'Angelo J Posner GH Yolken R. *In vitro* inhibition of *Toxoplasma gondii* by four new derivatives of artemisinin. Antimicrob Agents Chemother. (2006) 50:4206–8. 10.1128/AAC.00793-06 17060514 PMC1693994

[30] Wahab T Edvinsson B Palm D Lindh J. Comparison of the AF146527 and B1 repeated elements, two real-time PCR targets used for detection of *Toxoplasma gondii*. J Clin Microbiol. (2010) 48:591–2. 10.1128/JCM.01113-09 19940050 PMC2815584

[31] Del L Yácono M Farran I Becher ML Sander V Sánchez VR Martín V. A chloroplast-derived *Toxoplasma gondii* GRA4 antigen used as an oral vaccine protects against toxoplasmosis in mice. Plant Biotechnol. J. (2012) 10:1136–44. 10.1111/pbi.12001 23020088

[32] Albarracín RM Becher ML Farran I Sander VA Corigliano MG Yácono ML The fusion of *Toxoplasma gondii* SAG1 vaccine candidate to *Leishmania infantum* heat shock protein 83-kDa improves expression levels in tobacco chloroplasts. Biotechnol J. (2015) 5:748–59. 10.1002/biot.201400742 25823559

[33] Chang CL Chung CY Kuo CH Kuo TF Yang CW Yang WC Beneficial effect of *Bidens pilosa* on body weight gain, food conversion ratio, gut bacteria and coccidiosis in chickens. PLoS ONE. (2016) 1:e0146141 10.1371/journal.pone.0146141 PMC4713076 26765226

[34] Zoschke R Nakamura M Liere K Sugiura M Börner T Schmitz-Linneweber C. An organellar maturase associates with multiple group II introns. Proc Natl Acad Sci USA. (2010) 107:3245–50. 10.1073/pnas.0909400107 20133623 PMC2840290

[35] Hao da C Chen SL Xiao PG. Molecular evolution and positive Darwinian selection of the chloroplast maturase matK. J Plant Res. (2010) 123:241–7. 10.1007/s10265-009-0261-5 19943076

[36] Belfort M. Two for the price of one: a bifunctional intron-encoded DNA endonuclease-RNA maturase. Genes Dev. (2003) 17:2860–3. 10.1101/gad.1162503 14665667

[37] Nouioui I Ghodhbane-Gtari F Fernandez MP Boudabous A Normand P Gtari M. Absence of cospeciation between the uncultured Frankia microsymbionts and the disjunct actinorhizal Coriaria species. Biomed Res Int. (2014) 2014:924235. 10.1155/2014/924235 24864264 PMC4016943

[38] Badgujar SB Mahajan RT. Peptide mass fingerprinting and N-terminal amino acid sequencing of glycosylated cysteine protease of *Euphorbia nivulia Buch.-Ham.* J Amino Acids. (2013) 2013:569527. 10.1155/2013/569527 23476742 PMC3588393

[39] Fišer-Pečnikar Z Buzan EV. 20 years since the introduction of DNA barcoding: from theory to application. J Appl Genet. (2014) 55:43–52. 10.1007/s13353-013-0180-y 24203863

[40] McFadden GI. The apicoplast. Protoplasma. (2011) 248:641–50. 10.1007/s00709-010-0250-5 21165662

