# Supplementary figures and images for: Acetonic Fraction of Bidens pilosa Enriched for Maturase K Is Able to Control Cerebral Parasite Burden in Mice Experimentally Infected With Toxoplasma gondii
Source: Front Vet Sci. 2019 Mar 6;6:55. doi: 10.3389/fvets.2019.00055 (PMC6414801; doi:10.3389/fvets.2019.00055)

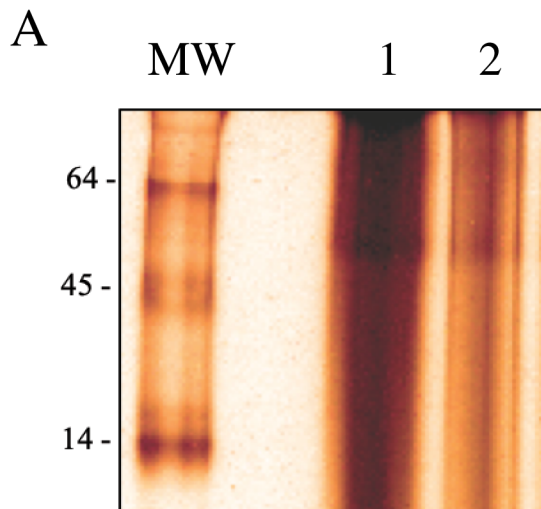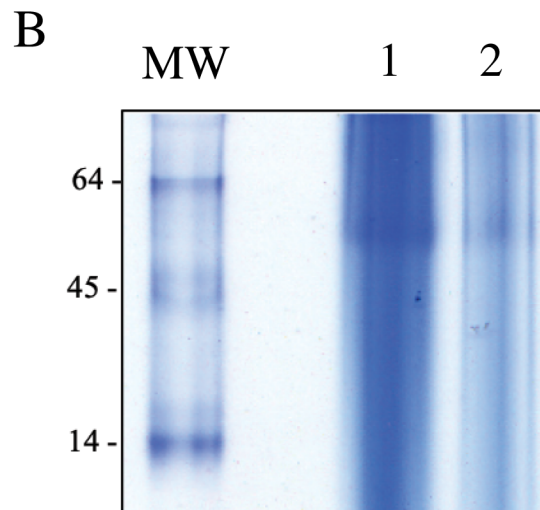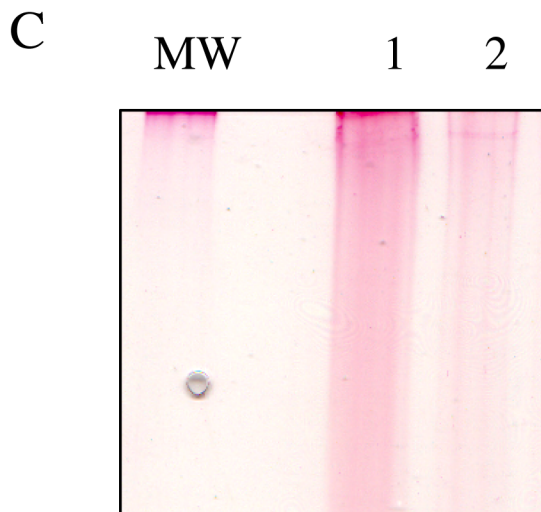

Supplement: Supplementary Figure 1S — One-dimensional gel electrophoresis (1-DE), resolved by 12% SDS-PAGE and stained by silver stain (A) or native gel (B) stained by Coomassie brilliant blue G-250® or periodic acid-Schiff—PAS (C). MW, molecular weight markers; 1, total extract of B. pilosa; 2, acetonic fraction of B. pilosa. Arrow—component identified as maturase k in the acetonic fraction from Bidens pilosa. [file Image_2.pdf]
