# Supplementary material for: Acetonic Fraction of Bidens pilosa Enriched for Maturase K Is Able to Control Cerebral Parasite Burden in Mice Experimentally Infected With Toxoplasma gondii
Source: Front Vet Sci. 2019 Mar 6;6:55. doi: 10.3389/fvets.2019.00055 (PMC6414801; doi:10.3389/fvets.2019.00055)
Supplement: Supplementary Table 1S — Analyses of Mass Spectrometry (MS) of Bidens pilosa acetonic fraction from two-dimensional and one-dimensional gel by NCBI Database (https://www.ncbi.nlm.nih.gov). [file Image_1.pdf]

Supplementary Table 1S: Mass Spectrometry (MS) analyses of the *Bidens pilosa* acetonic fraction from two-dimensional and one-dimensional gel by NCBI Databases (https://www.ncbi.nlm.nih.gov)

**1. MS Analyses: *Bidens pilosa* (taxid:42337) / Asteraceae (taxid:4210)**	
**Sequence**	**Name**
(R)IPYPTLGNK(N)	maturase K [matk]
(K)ALVSSIPR(E)	maturase K [matk]
(R)ISQVINSER(T)	maturase K [matk]
**2. MS Analyses: *Bidens pilosa* (taxid:42337)**	
**Sequence**	**Name**
(R)NIGIMAHIDAGKTTTTER(I)	maturase K [matk]
(K)KAVANASPVLLEPIMR(V)	maturase K [matk]
(K)AVANASPVLLEPIMR(V)	maturase K [matk]

[1] Mass spectrometry of two-dimensional (2-DE) gel spot;

[2] Mass spectrometry of One-dimensional (1-DE) gel band.
